# Fate of chlorpyrifos, omethoate, cypermethrin, and deltamethrin during wheat milling and Chinese steamed bread processing

**DOI:** 10.1002/fsn3.1523

**Published:** 2021-05-01

**Authors:** Lili Yu, Huijie Zhang, Xinning Niu, Li Wu, Yan Zhang, Bujun Wang

**Affiliations:** ^1^ Chinese Academy of Agricultural Sciences / Laboratory of Quality and Safety Risk Assessment for Cereal Products (Beijing) Institute of Crop Sciences Ministry of Agriculture Beijing China

**Keywords:** Chinese steamed bread, chlorpyrifos, cypermethrin, deltamethrin, omethoate

## Abstract

To investigate the fractioning of chlorpyrifos, omethoate, cypermethrin, and deltamethrin during wheat milling and the fate of four pesticides during Chinese steamed bread (CSB) processing, wheat samples, which were sprayed twice with chlorpyrifos, omethoate, cypermethrin, and deltamethrin at three levels of concentrations during the grain‐filling stage, were milled, and wheat flour was processed to CSB. The residues of four pesticides in the milling products, kneaded dough, fermented dough, and CSB were determined with GC‐MS/MS. The concentrations of chlorpyrifos, omethoate, cypermethrin, and deltamethrin in bran were 1.46–1.57, 1.85–2.13, 1.27–1.86, and 1.63–2.33 times higher than those in wheat, respectively, while the residues of the four pesticides in shorts decreased approximately 27.97% to 57.02% for chlorpyrifos, 6.22% to 44.77% for cypermethrin, and 13.13% to 61.15% for deltamethrin compared with the residues in wheat (*p* < .05); however, omethoate levels approximately doubled in the ten‐fold treatment group in shorts compared with those in wheat (*p* < .05). The residues of the four pesticides in flour were significantly lower than those in wheat, ranging from 38.68% to 98.04%. Chlorpyrifos and omethoate levels showed a slight decrease during the kneading and fermentation process, and further decreases of 2.46%–29.51% for chlorpyrifos and 14.22%–71.11% for omethoate were found in CSB; however, most of the groups of cypermethrin and deltamethrin showed various degrees of increases in kneaded and fermented dough and steamed bread compared with flour. The mechanism of this increase is unknown and needs further research.

## Graphical abstract

Milling process decreased chlorpyrifos, omethoate, cypermethrin, and deltamethrin levels in flour. Cypermethrin and deltamethrin levels showed various degrees of increases during Chinese steamed bread processing. Changes of chlorpyrifos, omethoate, cypermethrin, and deltamethrin during Chinese steamed bread making process were firstly detailed reported.

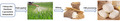

## 1 INTRODUCTION

Wheat is the second largest grain crop in China. Many kinds of pesticides are used yearly to control pests and diseases that threaten wheat yield and quality. Hence, pesticides play a major role in controlling pests and diseases, improving crop yield and quality, and ensuring the quality and safety of agricultural products (Zhao et al., 2014; Zhou & Jin, 2009); however, with the widespread use of pesticides, its adverse effects have become increasingly prominent. Pesticides and their degradation and metabolites can cause pollution not only to the crop products but also to the ecological environment, such as soil, air, and water, thus damaging the ecological balance. These pollutants may enter and accumulate in the human body through the food chain, thus endangering human health.

Chlorpyrifos and omethoate are organophosphorus pesticides used during cultivation to protect wheat from pest and disease infestation, and cypermethrin and deltamethrin are two commonly used pyrethroids pesticides applied in stored wheat grain for the control of pests. Organophosphorus and pyrethroids pesticides are widely used in agriculture and public health due to their high efficiency, long‐term stability, rapidity, and broad spectrum (Liu et al., 2007). Organophosphorus and pyrethroid pesticides, however, can be absorbed by humans and animals through the skin, digestive tract, or respiratory tract (Liu, Liu, Liu, & Liu, 2010) and may cause acute and chronic toxicity, including neurotoxicity (Alves, Symington, Lee, & Clark, 2010; Shiozaki et al., 1999; Singh et al., 2012), immunotoxicity (Suwanchaichinda, Khamkong, & Satayavivad, 2005; Zabrodskii & Germanchuk, 2001), and reproductive toxicity (Ahmad, Hussain, & A., 2009; Elbetieha, Da'As, & Darmani, 2001; Recio et al., 2001).

To ensure the safe use of pesticides, relative regulations have been established throughout the world. The maximum residue limits (MRLs) of wheat have been established by many countries and organizations, such as the European Union (EU), Codex Alimentarius Commission (CAC), and USA. In EU, Australia, USA, Japan, and CAC, the MRLs of chlorpyrifos in wheat are 0.05, 0.1, 0.5, 0.5, and 0.5 mg/kg, respectively; the MRLs of cypermethrin in wheat are 2, 0.2, 0.2, 0.2, and 0.2 mg/kg, respectively; and the MRLs of deltamethrin in wheat are 2, 2, 1, 2, and 2 mg/kg, respectively. The MRLs of omethoate in wheat are 0.01, 0.05, and 0.1 mg/kg in the European Union (EU), Australia, and Japan, respectively (Kim, Huang, Zhu, & Patricia, 2009; Liu et al., 2010, 2018), while omethoate is regarded as a pesticide that cannot be detected in wheat in the United States (USA) and at the Joint FAO/WHO Meeting on Pesticide Residues (JMPR) based on the Codex Alimentarius Commission (CAC). In China, the MRLs for chlorpyrifos, omethoate, cypermethrin, and deltamethrin as pesticides in wheat are 0.5, 0.02, 0.2, and 0.5 mg/kg, respectively (2016). Although MRLs are reliable means for enforcing the acceptable use of pesticides, they do not permit the human health risk assessment from residue intake unless accurate knowledge about the fate of residues in wheat processing and the distribution of residues in the final wheat products is available (Fleurat‐Lessard, Chaurand, Marchegay, & Abecassis, 2007). Moreover, there are currently no MRL standards for pesticide residues in postharvest wheat samples including wheat milling samples and final wheat products. It is, therefore, of great research significance to understand the transfer mechanisms of pesticide residues during wheat milling, as well as further processing of wheat products.

Chinese steamed bread (CSB), a traditional staple food, has been consumed for nearly 2,000 years in China, representing more than 40% of Chinese wheat consumption (He, Liu, Javier Peña, & Rajaram, 2003). Generally, the manufacturing process of CSB begins with the mixing of wheat flour, water, and yeast, followed by fermentation (38°C) and steaming (100°C) (Liu et al., 2018). CSB is regarded as a healthy food due to the absence of toxic Maillard reaction products, such as acrylamide and furan, as well as the lower oil and sodium contents. Furthermore, the lower steaming temperature (100°C) during CSB processing may better maintain various endogenous and exogenous nutrients compared with baked bread (Zhu, 2014).

Little research has been reported on the fate of pesticide residues during the CSB‐making process. The objective of this study was to investigate the dissipation regularity of chlorpyrifos, omethoate, cypermethrin, and deltamethrin during wheat milling and the CSB‐making process, which will contribute to the establishment of MRLs in wheat products and the risk assessment of wheat‐based food consumers to residues.

## 2 MATERIALS AND METHODS

### 2.1 Chemicals and reagents

Pesticide standard solutions of chlorpyrifos, omethoate, deltamethrin, and cypermethrin (are all 1,000 mg/L) were obtained from Agro‐Environment Protection Institute, Ministry of Agriculture (Beijing, China). The commercial pesticide chlorpyrifos (45% emulsifiable concentrate [EC]) was purchased from the Jiangsu Jiahui Chemical Co., Ltd.; the commercial pesticide omethoate (40% EC) was purchased from Shandong Dongtai Agrochemicals Co., Ltd.; the commercial pesticide cypermethrin (45% EC) was purchased from Shandong Zhongshi Pharmaceutical Co., Ltd.; and the commercial pesticide deltamethrin (25 g/L EC) was purchased from Bayer CropScience Co., Ltd. Organic solvents, including acetonitrile, formic acid, and n‐hexane, used for both sample extraction and analysis, were of HPLC grade and purchased from Thermo Fisher Scientific Corporation. UPLC‐grade acetone was purchased from MREDA Technology, Inc. Ultrapure water was obtained from a Milli‐Q system (Millipore).

### 2.2 Preparation of standard solutions

The standard stock solutions of chlorpyrifos, omethoate, cypermethrin, and deltamethrin (100 mg/L) were diluted with acetone and stored at −20°C. Working standard solutions of chlorpyrifos, omethoate, cypermethrin, and deltamethrin (0.001, 0.02, 0.05, 0.1, 0.2, 0.5, and 1 mg/L) were prepared by diluting the stock solution. Correspondingly, matrix‐matched standard solutions of chlorpyrifos, omethoate, cypermethrin, and deltamethrin (0.001, 0.02, 0.05, 0.1, 0.2, 0.5, and 1 mg/L) were prepared by diluting the working matrix standard solutions. These solutions were stored in the darkness at 4°C, and the working standard solutions were not observed to degrade over 3 months.

### 2.3 Wheat sample preparation

A wheat field at Shunyi Farm, located in the northeast of Beijing, China (E116°33’, N40°13’), was divided into control plot, which was sprayed with water, and treatment plots, which were sprayed with 4 kinds of pesticides at 3 concentrations. Plot #1 was used as the control. Plots #2‐#5 were sprayed with commercial pesticides chlorpyrifos 45% EC, omethoate 40% EC, deltamethrin 25 g/L EC, and cypermethrin 45% EC at the recommended dosage (450 ml/hectare for chlorpyrifos, 400 ml/hectare for omethoate, 675 ml/hectare for cypermethrin, and 600 ml/hectare for deltamethrin). Plots #6‐#9 were sprayed with the 4 pesticides at twofold the recommended dosage and plots #10‐#13 at ten‐fold the recommended dosage. Each plot was sprayed twice at 14 and 7 d before harvest. Plots were randomly arranged, and each treatment was replicated 3 times. Wheat samples were harvested, placed in polyethylene bags and stored in a −40°C freezer.

### 2.4 Milling process

All wheat samples were wetted to 16.5% moisture content and left overnight before milling. Milling was carried out by a Chopin CD1 Laboratory Mill (Chopin, France) with two breaks and one stripping system. Three fractions, including bran, shorts, and flour, as well as wheat grain, were obtained and submitted for analyses of pesticide residues.

### 2.5 Chinese streamed bread (CSB) processing

Chinese streamed bread was prepared according to the Chinese Standard (procedure 10139‐93, Appendix A, 1993). The basic formula for making CSB consisted of 100 g of milled flour, 48 ml of distilled warm water (38°C), and 1 g of active dry yeast. The ingredients were mixed evenly by hand for 3 min, and then, dough was fermented in a fermentation cabinet (38°C, 85% RH) for 1 hr. After that, it was kneaded and shaped manually for 5 min into a round dough piece with a smooth surface. After resting at room temperature for 15 min, the dough was put in a steamer when the water was boiling and was steamed (100°C) for 20 min, followed by cooling at room temperature for 40–60 min. Three replicate samples at every stage (kneading, fermenting, and steaming) were taken. All representative samples were stored at −20°C until analysis.

### 2.6 Sample extraction and cleaning up

The extraction and cleaning up procedure was carried out following the QuEChERS method. Five grams of homogenized sample were weighted in a 50‐mL polypropylene centrifuge tube and then extracted with 20 ml of acetonitrile (50:50, *v*/*v*) for 30 min using an automatic shaker. Afterward, 4 g of magnesium sulfate (MgSO_4_), 1 g of sodium chloride (NaCl), 1 g of sodium citrate dihydrate, and 0.5 g of sodium hydrogen citrate sesquihydrate were added and shaken vigorously for 2 min, and the sample was centrifuged for 5 min at 6,037 *g*. Then, a clean‐up, dispersive, solid‐phase extraction (d‐SPE) was conducted by adding 5 ml of the supernatant phase to a 15‐ml centrifuge tube that contained 900 mg of MgSO_4_, 150 of mg PSA, and 150 of mg C_18_. The sample was immediately vortexed for 1 min and centrifuged for 5 min at 6,037 *g*, and then, 2 ml of the supernatant‐cleaned extract was evaporated to dryness in a nitrogen evaporator with a water bath at 60°C. The dry residue was then dissolved in 1 ml of acetone, followed by filtering through a 0.22‐μm nylon syringe filter (Jinteng). After that, it was ready for analysis.

### 2.7 Pesticide determination by GC‐MS/MS

Analyses of the pesticides were performed by a Bruker 450 GC system, equipped with an autosampler (Bruker CP 8400), that was coupled to a triple quadrupole mass spectrometer (Bruker 300 MS), operating in the electron impact ionization (EI) mode. The instrument conditions are as follows: the column was initially maintained at 60°C, and then, the temperature was increased to 250°C at a rate of 20°C/min and finally increased to 300°C at a rate of 5°C/min and maintained for 4.5 min. The total run time was 24 min. Argon collision gas flow was maintained at 1.8 m Torr pressure. Helium (purity > 99.999%) was employed as a carrier gas at 1.2 ml/min, and injections were carried out in split/splitless mode at 250°C using 1‐μL injection volumes.

### 2.8 Method validation

Recovery experiments were conducted by spiking untreated wheat samples at five different levels of 0.02, 0.05, 0.1, 0.2, and 0.5 mg/kg with intermediate pesticide working solutions (mixture of chlorpyrifos, omethoate, cypermethrin, and deltamethrin) in acetone. Triplicates of each concentration were analyzed. The limits of detection (LODs) and limits of quantitation (LOQs) for the four pesticides were assessed at a signal‐to‐noise (S/N) ratio of 3 and 10, respectively.

### 2.9 Statistical analysis

Statistical analysis was performed using the PSAW Statistic 19.0 (SPSS) statistical software package. All data were subjected to a one‐way analysis of variance (one‐way ANOVA). Homogeneity of variance was confirmed before ANOVA, and differences among the means were analyzed by Duncan's multiple range test. Data were shown and analyzed as micrograms per kilogram of matrix (µg/kg) on a dry matter basis. The differences were regarded as significant when *p* < .05, and data were reported as the mean value ± the standard deviation (*SD*) of five replicates.

The processing factor (PF) during different wheat milling fractions and CSB processing is calculated as follows: PF = C_1_/C_2_, where C_1_ and C_2_ are postprocessing and preprocessing pesticide residue levels (mg/kg), respectively.

## 3 RESULTS AND DISCUSSION

### 3.1 Method validation

The methods for the determination of chlorpyrifos, omethoate, cypermethrin, and deltamethrin residues in wheat grain, flour, bran, shorts, dough, and steamed bread using GC‐MS/MS were validated by spiking a series of the pesticide standard solutions to the wheat products. Table 1 summarizes the retention time, formula, qualitative ion, quantitative ion, and collision energy of omethoate, chlorpyrifos, cypermethrin, and deltamethrin obtained using GC‐MS/MS. The correlation coefficients (*R* ^2^), which show the correlation between the concentrations of pesticide residues and the detective areas in different wheat matrices, were higher than 99.10%, demonstrating that the methods were sensitive and selective (Table 2). Mean recoveries obtained ranged from 70.33% to 118.33% with a relative standard deviation (RSD) lower than 8.78%. The LOD for the four pesticides in different matrices ranged from 0.25 to 1.60 µg/kg, and the LOQ ranged from 0.80 to 5.00 µg/kg, which were below the maximum residue limits (MRLs) established by the EU, USA, and China.

**Table 1:** GC‐MS/MS accurate mass measurements of omethoate, chlorpyrifos, cypermethrin, and deltamethrin

Compound	Retention time (min)	Formula	Qualitative ion	Quantitative ion	Collision energy (eV)
Omethoate	7.24	C_2_H_7_O_4_P	156	110/141	10/10
Chlorpyrifos	9.34	C_9_H_11_Cl_3_NO_3_PS	314	258/286	15/5
Cypermethrin	15.39	C_22_H_19_Cl_2_NO_3_	163	91/127	20/10
Deltamethrin	17.51	C_22_H_19_Br_2_NO_3_	253	93/174	20/13

**Table 2:** *R* ^2^, recoveries, LOD, and LOQ of chlorpyrifos, omethoate, cypermethrin, and deltamethrin in different matrices

Pesticides	Matrix	*R* ^2^	Average recovery and standard deviations (%)					LOD (µg/kg)	LOQ (µg/kg)
			Spiking level (µg/kg)						
			20	50	100	200	500		
Chlorpyrifos	Whole wheat	0.9998	85.0 ± 5.0	90.7 ± 6.1	92.0 ± 3.6	90.3 ± 4.2	89.1 ± 4.2	0.25	0.80
	Flour	0.9981	107.7 ± 3.1	93.3 ± 2.3	91.3 ± 3.5	91.7 ± 2.9	89.1 ± 0.6	0.32	0.90
	Bran	0.9999	100.0 ± 2.0	88.7 ± 3.1	88.0 ± 2.0	75.2 ± 0.3	70.3 ± 1.8	0.35	1.00
	Shorts	0.9979	78.3 ± 2.9	74.0 ± 3.5	80.3 ± 2.3	82.5 ± 1.0	87.9 ± 3.2	0.30	0.90
	Dough	0.9986	98.3 ± 2.9	92.7 ± 3.1	94.3 ± 0.6	94.2 ± 2.8	92.6 ± 1.4	0.28	1.00
	Chinese steamed bread	0.9995	102.7 ± 2.5	93.3 ± 4.2	86.3 ± 1.2	77.3 ± 0.8	80.9 ± 0.7	0.20	0.80
Omethoate	Whole wheat	0.9996	76.7 ± 7.6	84.7 ± 7.0	79.3 ± 3.5	92.0 ± 4.0	94.9 ± 2.0	0.64	2.00
	Flour	0.9975	96.3 ± 8.1	85.3 ± 2.3	91.3 ± 3.1	90.8 ± 4.6	94.9 ± 2.0	0.70	2.20
	Bran	1.0000	115.0 ± 4.4	101.3 ± 3.1	97.3 ± 3.2	78.5 ± 1.3	77.9 ± 3.9	0.85	2.60
	Shorts	0.9972	93.3 ± 2.9	74.7 ± 3.1	79.7 ± 1.5	81.0 ± 1.3	87.0 ± 3.0	0.79	2.50
	Dough	0.9913	70.8 ± 5.2	75.0 ± 3.0	94.5 ± 1.8	109.3 ± 3.0	90.0 ± 2.8	0.80	2.50
	Chinese steamed bread	0.9980	111.7 ± 2.9	74.7 ± 3.1	72.7 ± 3.5	76.3 ± 1.6	91.7 ± 1.7	0.85	2.80
Cypermethrin	Whole wheat	0.9998	80.8 ± 4.9	83.6 ± 1.8	89.8 ± 2.0	94.7 ± 2.9	94.9 ± 1.8	1.36	5.00
	Flour	0.9997	110.8 ± 3.9	83.6 ± 1.8	88.1 ± 1.9	93.4 ± 4.5	96.9 ± 1.8	1.50	4.50
	Bran	0.9994	107.8 ± 1.6	75.8 ± 2.0	83.0 ± 2.8	72.5 ± 1.8	75.9 ± 2.6	1.60	5.00
	Shorts	1.0000	104.2 ± 3.0	70.8 ± 2.3	77.3 ± 4.5	85.1 ± 1.8	96.1 ± 3.7	1.05	3.50
	Dough	0.9996	100.8 ± 7.8	92.2 ± 3.0	93.4 ± 2.6	96.8 ± 2.9	116.5 ± 1.4	1.30	4.50
	Chinese steamed bread	0.9993	97.0 ± 7.2	80.8 ± 0.8	75.4 ± 2.3	80.0 ± 1.2	94.5 ± 2.1	1.50	5.00
Deltamethrin	Whole wheat	0.9991	78.3 ± 4.2	72.7 ± 5.0	75.7 ± 2.1	88.8 ± 4.5	93.3 ± 1.6	0.35	1.00
	Flour	0.9939	110.0 ± 7.6	76.7 ± 5.0	73.7 ± 1.5	92.3 ± 4.9	112.0 ± 1.6	0.30	0.90
	Bran	0.9920	103.3 ± 2.9	73.9 ± 7.3	72.7 ± 3.1	80.3 ± 2.5	74.2 ± 2.0	0.25	0.80
	Shorts	0.9940	103.3 ± 7.4	82.7 ± 1.2	72.0 ± 1.0	77.2 ± 1.9	101.1 ± 4.0	0.30	1.00
	Dough	0.9942	110.0 ± 8.7	86.0 ± 6.9	84.7 ± 3.5	88.3 ± 8.8	108.1 ± 5.7	0.28	0.90
	Chinese steamed bread	0.9946	118.3 ± 2.9	77.3 ± 6.1	74.0 ± 3.6	75.0 ± 5.2	89.7 ± 3.4	0.30	1.00

### 3.2 Fate of chlorpyrifos, omethoate, cypermethrin, and deltamethrin during wheat milling

Accurate knowledge about the fractioning of pesticide residues during the wheat milling process is important for the establishment of MRLs in grains and wheat‐based food risk assessment. Many previous studies have indicated that most pesticide residues remained on the surface of grain and only a small part penetrated to the internal portions of wheat, and consequently residue levels in brain were consistently higher than that in wheat (Holland, Hamilton, Ohlin, & Skidmore, 1994; Kaushik, Satya, & Naik, 2009; Uygun, Koksel, & Atli, 2005; Uygun, Senoz, & Hamit, 2008). Malathion and fenitrothion were applied to wheat at toxicant concentrations of 8.89 and 24.50 mg/kg before processing into flour. During flour‐making, only trace amounts (7.2% and 9.6% residues) were found in the flour, and levels of malathion and fenitrothion in bran can be 1.1 and 1.4 times greater than in wheat, respectively (Uygun et al., 2005). Sgarbiero, Baptista, and Trevizan (Sgarbiero, Baptista, & Trevizan, 2002) demonstrated that 12 mg/kg pirimiphos‐methyl was added into wheat grain and stored for 240 days. After the milling process, the pirimiphos‐methyl level in bran was approximately 2.5 times that in wheat, but 60% of the residues were still found in white flour. Joia, Webster, and Loschiavo (Joia, Webster, & Loschiavo, 1985) reported that the highest concentrations of cypermethrin and fenvalerate were accumulated in bran, and the least, in endosperm. Reductions of 79%–84% for cypermethrin and 87%–88% for fenvalerate were present in flour.

As expected, in our study, chlorpyrifos, omethoate, cypermethrin, and deltamethrin levels varied significantly among the milling products (*p* < .05), in which the concentrations of the four pesticides in bran were significantly enhanced (*p* < .05), and in flour, they were significantly lower than in wheat (*p* < .05) (Table 3). The concentrations of chlorpyrifos, omethoate, cypermethrin, and deltamethrin in bran were 1.46–1.57, 1.85–2.13, 1.27–1.86, and 1.63–2.33 times higher than that in wheat, respectively. Obviously, the PF values of four pesticides were all >1 during the malting process (Table 4). Meanwhile, the residues of the pesticides in shorts decreased approximately 27.97% to 57.02% for chlorpyrifos, 6.22% to 44.77% for cypermethrin, and 13.13% to 61.15% for deltamethrin compared with residues in wheat (*p* < .05); however, the omethoate showed a different trend with a marked increase in shorts compared with in wheat (*p* < .05) in the ten‐fold treatment group due to its strong systemic conductibility. Further degradation of four pesticide levels was found in flour, with ranges of an 85.45%–89.67% decrease in chlorpyrifos, 85.68%–98.04% decrease in omethoate, 38.68%–83.69% decrease in cypermethrin, and 63.10%–93.65% decrease in deltamethrin. Additionally, the PF values of chlorpyrifos, cypermethrin, and deltamethrin were <1 in shorts and PF values of four pesticide residues decreased to 0.02–0.61 in flour (Table 4), which indicated that milling process affects the removal of pesticide residues significantly.

**Table 3:** Pesticide residue levels in different wheat milling fractions (ug/kg)

Sample matrix	Chlorpyrifos			Omethoate			Cypermethrin			Deltamethrin		
	Treatment 1	Treatment 2	Treatment 3	Treatment 1	Treatment 2	Treatment 3	Treatment 1	Treatment 2	Treatment 3	Treatment 1	Treatment 2	Treatment 3
Wheat	591.1 ± 5.5^c^	1,181.2 ± 33.4^c^	4,459.8 ± 77.5^c^	180.1 ± 8.4^c^	333.8 ± 22.3^b^	1,208.7 ± 22.4^b^	53.2 ± 2.2^c^	131.2 ± 1.7^c^	709.2 ± 2.8^c^	18.7 ± 1.1^b^	72.6 ± 2.2^c^	806.8 ± 3.4^c^
Bran	895.1 ± 44.2^d^	1855.3 ± 83.8^d^	6,520.4 ± 300.2^d^	333.9 ± 12.8^d^	710.2 ± 25.7^c^	2,495.7 ± 9.6^d^	67.4 ± 1.5^d^	240.4 ± 4.4^d^	1,317.7 ± 39.1^d^	43.6 ± 1.8^d^	118.5 ± 4.4^d^	1,476.2 ± 10.2^d^
Shorts	425.8 ± 11.2^b^	815.0 ± 9.0^b^	1916.8 ± 8.8^b^	92.7 ± 5.4^b^	359.7 ± 6.0^b^	2,145.4 ± 54.3^c^	49.9 ± 0.5^b^	86.6 ± 4.8^b^	391.7 ± 16.9^b^	16.3 ± 1.7^c^	45.8 ± 2.4^b^	313.4 ± 17.9^b^
Flour	61.0 ± 2.8^a^	171.8 ± 4.2^a^	573.2 ± 4.8^a^	3.5 ± 0.4^a^	35.3 ± 3.2^a^	173.0 ± 3.6^a^	32.6 ± 0.9^a^	37.6 ± 1.3^a^	115.7 ± 1.6^a^	1.2 ± 0.0^a^	26.8 ± 1.4^a^	91.2 ± 2.4^a^

Note

Values (mean ± *SD*) in the same row with different superscript letters are significantly different (*p* < .05).

Treatment 1: Recommended dosage.

Treatment 2: Twofold of the recommended dosage.

Treatment 3: Ten‐fold of the recommended dosage.

**Table 4:** Processing factors during different wheat milling fractions and CSB processing

Sample matrix	Chlorpyrifos			Omethoate			Cypermethrin			Deltamethrin		
	Treatment 1	Treatment 2	Treatment 3	Treatment 1	Treatment 2	Treatment 3	Treatment 1	Treatment 2	Treatment 3	Treatment 1	Treatment 2	Treatment 3
Bran	1.51	1.57	1.46	1.85	2.13	2.07	1.27	1.83	1.86	2.33	1.63	1.83
Shorts	0.72	0.69	0.43	0.51	1.08	1.77	0.94	0.66	0.55	0.87	0.63	0.39
Flour	0.10	0.15	0.13	0.02	0.11	0.14	0.61	0.29	0.16	0.06	0.37	0.11
Kneaded dough	0.10	0.12	0.13	—	0.14	0.13	0.79	0.34	0.17	—	0.54	0.13
Fermented dough	0.10	0.12	0.12	—	0.24	0.11	1.17	0.48	0.16	—	1.03	0.15
Steamed dough	0.10	0.10	0.10	—	0.09	0.04	0.76	0.33	0.14	—	0.53	0.09

High concentrations of pesticides accumulated in the bran fraction may be related to the strength of fixation of residues on the bran coat. The thicker of the bran layers and higher yield of bran may have led to more residues accumulated on the bran coat. The transfer of pesticide residues to bran, shorts, and flour fractions can also be attributed to a translocation phenomenon generated by mechanical power during the milling process. Residues are not completely absorbed into the bran layer and can still be easily removed and transferred to the other layers (shorts and flours) through amplified friction, crushing, rolling, or sieving steps during the milling process (Fleurat‐Lessard et al., 2007). Furthermore, milling temperature and time also have an impact on the degradation of pesticides. They can be in interaction with the mechanical power during milling operations, which then leads to the structural changes of pesticides, thereby resulting in the degradation of pesticides in the final product.

### 3.3 Fate of chlorpyrifos, omethoate, cypermethrin, and deltamethrin during CSB processing

**Figure 1:**
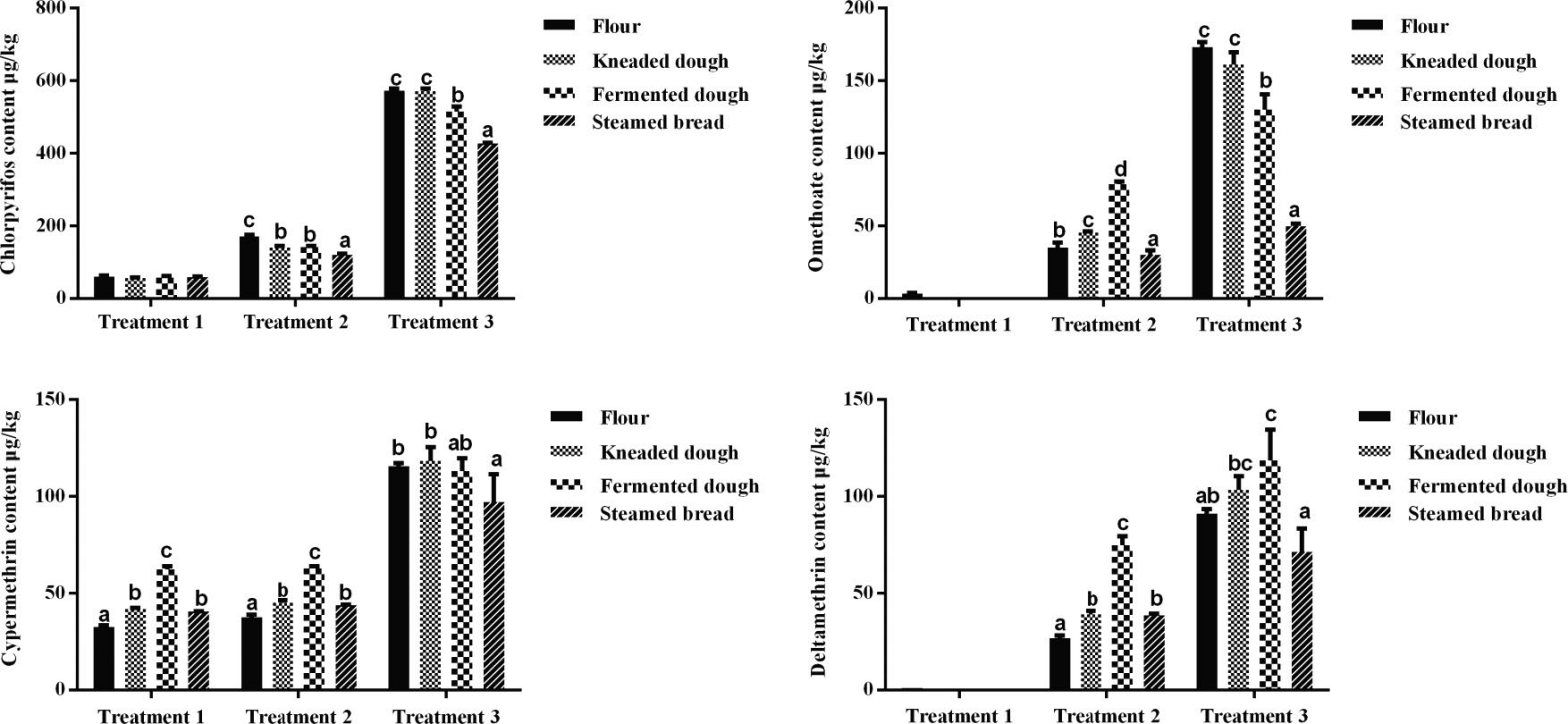
Pesticide residue levels during Chinese steamed bread (CSB) making process (µg/kg)

In general, it is accepted that most processing techniques, such as cleaning, peeling, milling, extruding, cooking, baking, canning, and juicing, might reduce the pesticide residues in food (Abou‐Arab, 1999; Sharma, Satya, Kumar, & Tewary, 2005; Soliman, 2001). In some special cases, however, some toxic metabolites and by‐products can be formed, and the pesticide residues could increase during food processing (Bajwa & Sandhu, 2014). In the present study, the flours mixed by the break and reduction flours after milling were prepared to make CSB. The normal CSB‐making process involves three major steps: kneading, fermentation, and steaming. As shown in Figure 1, the concentrations of chlorpyrifos, omethoate, cypermethrin, and deltamethrin showed significant changes compared with flour during the CSB‐making process. Omethoate, cypermethrin, and deltamethrin concentrations in the kneaded dough of most treatment groups increased significantly (*p* < .05) compared with flour, while chlorpyrifos concentration showed a slight decrease during the kneading process. This could be contributed to the fact that the insufficient mixing of the flour caused inconsistent concentrations among the samples. Samples from the recommended dosage of omethoate and deltamethrin were too little to quantify because of their lower initial levels in flour. Additionally, in this study, the increased pesticide concentrations after kneading were mostly found in the recommended dosage and twofold of the recommend dosage groups. Hence, it is suggested that the enhanced matrix interference resulted in increased recovery of pesticides in the low level, and this was in accordance with the results that are shown in Table 2, in which the lower spiking levels (20 µg/kg) displayed a higher average recovery in different matrices.

Yeast‐mediated fermentation is an essential process of wheat dough preparation in the traditional CSB‐making process (Kim et al., 2009). It has been reported that many factors could affect the fate of pesticides during food fermentation, such as chemical structure, volatility, and adsorption ability to matrices of pesticides, biodegradation of microorganisms, and cleavage of extracellular enzymes (Aislabie & Lloyd‐Jones, 1995; Azizi & A. Homayouni, 2009; Bayarri, Conchello, Ariño, Lázaro, & Herrera, 2015). These factors, themselves, are dependent on many environmental parameters, including processing temperature, moisture content, pH, and light (Aislabie & Lloyd‐Jones, 1995; Azizi & A. Homayouni, 2009). In the present study, we observed that the four pesticides showed different sensitivity to the applied dough fermentation. After fermentation (38°C, 1 hr), most groups of chlorpyrifos and omethoate showed lower pesticide concentrations compared with the flour (*p* < .05) (Figure 1). Similar results were reported by Sharma, Satya, Kumar, & Tewary (Sharma et al., 2005), in which yeast could degrade organophosphorus pesticides during the bread making progress, and yeast fermentation and baking removed 50.66%–88.35% of chlorpyrifos and 59.85%–79.04% of malathion compared with the wheat flour. Additionally, Zhou, Liu, and Zhao (2015) reported that organophosphorus pesticide concentrations decreased in ranges of 16.6%–26.6% to 23.4%–31.8% when added to 1.5% and 3.0% yeast after a fermentation time of 5 hr, depending on the fermentation time and yeast addition levels. These results proved that yeast had a clear ability to degrade some pesticides belonging to organophosphorus groups, and fermentation time played an important role in pesticide degradation. In our study, however, higher concentrations of cypermethrin and deltamethrin residues in fermented dough were observed than that in flour (*p* < .05). Obviously, pyrethroid groups showed a completely opposite trend compared with organophosphorus groups during fermentation, which might be partly governed by their chemical structure. Mao et al. (Mao et al., 2008) demonstrated that *Candida tropicalis* markedly affected the fate of organophosphorus pesticides, such as chlorpyrifos, dimethoate, isocarbophos, and triazophos (27.95%–62.28% reduction rates), during the fermentation of sweet orange peel, whereas no significant changes were observed in pyrethroid groups such as cypermethrin, deltamethrin, and bifenthrin during the fermentation process, indicating that the stability of pesticides might significantly affect the degradation rate of pesticides during fermentation. Furthermore, the observed increases in cypermethrin and deltamethrin levels in fermented dough are also likely due to a concentration effect or affinity for the lipid moiety of pesticides (Bajwa & Sandhu, 2014; Regueiro, López‐Fernández, Rial‐Otero, Cancho‐Grande, & Simal‐Gándara, 2015). Cypermethrin and deltamethrin are more strongly lipophilic pesticides than chlorpyrifos and omethoate, and they have strong affinities for the lipid moiety; thus, they are more easily combined with the lipid in flour during the CSB‐making process, resulting in increased pesticide concentrations in dough and the final product.

In our study, reductions of 2.46%–29.51% for chlorpyrifos and 14.22%–71.11% for omethoate were observed during the steaming process (100°C, 20 min) compared with the initial levels in flour (Figure 1), and higher reductions were found during steaming with higher initial pesticide levels in flour. The results agreed with Sharma, Satya, Kumar, & Tewary (Sharma et al., 2005), who spiked four different concentrations (1, 2, 3, and 4 mg/kg) in wheat flours with six pesticides to make bread and found that the range of pesticide degradation was 47%–89%. Pal and Shah (Pal & Shah, 2008) found 32.5%–72.4% chlorpyrifos reduction during the bread making process. When flour was converted into white bread, degradation of the malathion and fenitrothion residues was approximately 81%–85% and 61%–88%, respectively (Uygun et al., 2005). Further, other food processing, such as cookie and pasta processing, can also lead to a significant reduction in organophosphorus pesticides in the final product (Uygun et al., 2008; Uygun, Senoz, Öztürk, & Koksel, 2009). These findings give strong support to the present results, which suggest that the heating process can lead to a large reduction in pesticides, which might be influenced by evaporation, codistillation, or thermal degradation, as well as the physicochemical properties of the pesticides, including the volatility, solubility, and hydrolysis rate constant (Holland et al., 1994; Kaushik et al., 2009; Sharma et al., 2005). Moreover, omethoate residue levels in CSB of the twofold and ten‐fold treatment groups exceeded the MRLs (0.02 mg/kg), implying that the remaining omethoate in flour and CSB might endanger consumer health; thus, the use of omethoate in wheat should be strictly controlled. The cypermethrin and deltamethrin levels in CSB, however, were significantly higher than in flour (*p* < .05), increasing 1.16‐fold to 1.43‐fold compared to the flour, except for a decrease presented in the ten‐fold treatment group (loss of 16.41% for cypermethrin and 21.69% for deltamethrin) (Figure 1). Obviously, in this study, most cypermethrin and deltamethrin groups showed various degree of increase in dough and steamed bread compared with flour. To date, there is little information available that may explain this phenomenon, and the possible reasons might be (a) the great penetration of lipophilic pesticides into the grain and then migration from the seed coat into the endosperm during the CSB‐making process; (b) the thermal and hydrolytic stability of pyrethroid pesticides leading to a greater persistence of pesticides in the final products; and (c) the concentration effect in which the low concentration groups are more susceptible to matrix interference, resulting in increased recovery of pesticides. Further research needs to clarify the actual mechanism that occurs during the CSB‐making process.

## 4 CONCLUSION

This study investigated the fractioning and fate of chlorpyrifos, omethoate, cypermethrin, and deltamethrin during wheat milling and Chinese steamed bread processing. The levels of chlorpyrifos, omethoate, cypermethrin, and deltamethrin in bran were 1.46–1.57, 1.85–2.13, 1.27–1.86, and 1.63–2.33 times higher than those in wheat, respectively, while reductions of 27.97%–57.02% for chlorpyrifos, 6.22%–44.77% for cypermethrin, and 13.13%–61.15% for deltamethrin were observed in shorts compared with those in wheat (*p* < .05), with omethoate showing a different trend with a marked increase in the ten‐fold treatment group in shorts compared with that in wheat (*p* < .05). Decreases in the four pesticides ranging from 38.68% to 98.04% were observed in flour.

To the best of our knowledge, this is the first detailed report focusing on the fate of chlorpyrifos, omethoate, cypermethrin, and deltamethrin in wheat milling regarding Chinese steamed bread processing. The results showed that chlorpyrifos and omethoate levels slightly decreased during the kneading and fermentation processing, and further decreases of 2.46%–29.51% for chlorpyrifos and 14.22%–71.11% for omethoate were observed in CSB compared with flour. The omethoate residue levels in CSB of the twofold and ten‐fold treatment groups exceeded the MRLs (0.02 mg/kg). Although the concentrations of cypermethrin and deltamethrin in flour did not exceed the MRLs of China (0.2 and 0.5 mg/kg), most of the cypermethrin and deltamethrin groups showed various degrees of increases in kneaded and fermented dough and CSB compared with flour due to a concentration effect, thermal and hydrolytic stability and a strong affinity for the lipid moiety of cypermethrin and deltamethrin, which might also endanger human food safety. The research results provide further information for wheat‐based food risk assessment in terms of pesticide residues.

## CONFLICT OF INTEREST

There are no conflicts of interest.

## ETHICAL APPROVAL

This study does not involve any human or animal testing.

## References

[fsn31523-bib-0001] Abou‐Arab, A. K. (1999). Behavior of pesticides in tomatoes during commercial and home preparation. Food Chemistry, 65, 509–514.

[fsn31523-bib-0002] Ahmad, M. , Hussain, I. , Khan, A. , & Najib‐ur‐Rehman (2009). Khan and Najib‐ur‐Rehman, Deleterious effects of cypermethrin on semen characteristics and testes of dwarf goats (*Capra hircus*). Experimental and Toxicologic Pathology, 61, 339–346. 19019642 10.1016/j.etp.2008.10.002

[fsn31523-bib-0003] Aislabie, J. , & Lloyd‐Jones, G. (1995). A review of bacterial degradation of pesticides. Australian Journal of Soil Research, 33, 925–942.

[fsn31523-bib-0004] Alves, M. , Symington, S. B. , Lee, S. H. , & Clark, J. M. (2010). PKC‐dependent phosphorylations modify the action of deltamethrin on rat brain N‐type (CaV2.2) voltage‐sensitive calcium channel. Pesticide Biochemistry and Physiology, 97, 101–108.

[fsn31523-bib-0005] Azizi, A. , & Homayouni, A. (2009). Bacterial‐degradation of pesticides residue in vegetables during fermentation. Asian Journal of Chemistry, 21, 6255–6264.

[fsn31523-bib-0006] Bajwa, U. , & Sandhu, K. S. (2014). Effect of handling and processing on pesticide residues in food‐a review. Journal of Food Science and Technology, 51, 201–220. 24493878 10.1007/s13197-011-0499-5 PMC3907644

[fsn31523-bib-0007] Bayarri, S. , Conchello, P. , Ariño, A. A. , Lázaro, R. , & Herrera, A. (2015). Evaluation of an analytical method for an in‐vitro study of degradation of organochlorine compounds by ‘meat starter’ micro‐organisms. Pest Management Science, 50, 120–126.

[fsn31523-bib-0008] Elbetieha, S. I. , Da'As, W. K. , & Darmani, H. (2001). Evaluation of the toxic potentials of cypermethrin pesticide on some reproductive and fertility parameters in the male rats. Archives of Environmental Contamination and Toxicology, 41, 522–528. 11598791 10.1007/s002440010280

[fsn31523-bib-0009] Fleurat‐Lessard, F. , Chaurand, M. , Marchegay, G. , & Abecassis, J. (2007). Effects of processing on the distribution of pirimiphos‐methyl residues in milling fractions of durum wheat. Journal of Stored Products Research, 43, 384–395.

[fsn31523-bib-0010] He, Z. H. , Liu, A. H. , Javier Peña, R. , & Rajaram, S. (2003). Suitability of Chinese wheat cultivars for production of northern style Chinese steamed bread. Euphytica, 131, 155–163.

[fsn31523-bib-0011] Holland, P. T. , Hamilton, D. , Ohlin, B. , & Skidmore, M. (1994). Pesticides report 31: Effects of storage and processing on pesticide residues in plant products (Technical Report). Pure and Applied Chemistry, 66, 335–356.

[fsn31523-bib-0012] Joia, S. , Webster, G. R. B. , & Loschiavo, S. R. (1985). Cypermethrin and fenvalerate residues in stored wheat and milled fractions. Journal of Agriculture and Food Chemistry, 33, 618–622.

[fsn31523-bib-0013] Kaushik, G. , Satya, S. , & Naik, S. N. (2009). Food processing a tool to pesticide residue dissipation ‐ A review. Food Research International, 42, 26–40.

[fsn31523-bib-0014] Kim, Y. , Huang, W. , Zhu, H. , & Patricia, R. D. (2009). Spontaneous sourdough processing of Chinese Northern‐style steamed breads and their volatile compounds. Food Chemistry, 114, 685–692.

[fsn31523-bib-0015] Liu, P. Y. , Liu, Y. J. , Liu, Q. X. , & Liu, J. W. (2010). Photodegradation mechanism of deltamethrin and fenvalerate. Journal of Environmental Sciences, 22, 1123–1128. 10.1016/s1001-0742(09)60227-8 21175006

[fsn31523-bib-0016] Liu, T. J. , Li, Y. , Sadiq, F. A. , Yang, H. Y. , Gu, J. S. , Yuan, L. , … He, G. (2018). Predominant yeasts in Chinese traditional sourdough and their influence on aroma formation in Chinese steamed bread. Food Chemistry, 242, 404–411. 29037707 10.1016/j.foodchem.2017.09.081

[fsn31523-bib-0017] Liu, T. F. , Sun, C. , Ta, N. , Hong, J. , Yang, S. G. , & Chen, C. X. (2007). Effect of copper on the degradation of pesticides cypermethrin and cyhalothrin. Journal of Environmental Sciences, 19, 1235–1238. 10.1016/s1001-0742(07)60201-0 18062423

[fsn31523-bib-0018] Mao, X. F. , Jiao, B. N. , Qian, Y. Z. , Zhao, Q. Y. , Fu, C. M. , & Sun, Z. G. (2008). Effects of fermentation on pesticide residues in sweet orange peel. Food and Fermentation Industries, 34, 59–63. (in Chinese).

[fsn31523-bib-0019] National Standard Publishing House (2016). National standard of the People's Republic of China: GB 2763‐2016, The maximum residue limit of pesticides in food. Beijing: China Standard Publishing House. pp. 54–198.

[fsn31523-bib-0020] Pal, P. , & Shah, P. G. (2008). Effect of storage & processing on dissipation of five insecticides on wheat. Pesticide Research Journal, 20, 253–258.

[fsn31523-bib-0021] Recio, R. , Robbins, W. A. , Ocampo‐Gómez, G. , Borja‐Aburto, V. , Morán‐Martínez, J. , Froines, J. R. , … Cebrián, M. E. (2001). Organophosphorous pesticide exposure increases the frequency of sperm sex null aneuploidy. Environmental Health Perspectives, 109, 1237–1240. 11748030 10.1289/ehp.011091237 PMC1240505

[fsn31523-bib-0022] Regueiro, J. , López‐Fernández, O. , Rial‐Otero, R. , Cancho‐Grande, B. , & Simal‐Gándara, J. (2015). A review on the fermentation of foods and the residues of pesticides‐biotransformation of pesticides and effects on fermentation and food quality. Critical Reviews in Food Science and Nutrition, 55, 839–863. 24915365 10.1080/10408398.2012.677872

[fsn31523-bib-0023] Sgarbiero, E. , Baptista, G. C. D. , & Trevizan, L. R. P. (2002). Pirimiphos‐methyl residues in wheat grain and in some of its processed products. Revista Brasileira De Toxicologia, 15, 5–8. (in Portuguese).

[fsn31523-bib-0024] Sharma, J. , Satya, S. , Kumar, V. , & Tewary, D. K. (2005). Dissipation of pesticides during bread‐making. Chemical Health and Safety, 12, 17–22.

[fsn31523-bib-0025] Shiozaki, K. , Iseki, E. , Uchiyama, H. , Watanabe, Y. , Haga, T. , Kameyama, K. , … Kosaka, K. (1999). Alterations of muscarinic acetylcholine receptor subtypes in diffuse lewy body disease: Relation to Alzheimer's disease. Journal of Neurology, Neurosurgery and Psychiatry, 67, 209–213. 10.1136/jnnp.67.2.209 PMC1736504 10406992

[fsn31523-bib-0026] Singh, K. , Tiwari, M. N. , Upadhyay, G. , Patel, D. K. , Singh, D. , Prakash, O. , & Singh, M. P. (2012). Long term exposure to cypermethrin induces nigrostriatal dopaminergic neurodegeneration in adult rats: Postnatal exposure enhances the susceptibility during adulthood. Neurobiology of Aging, 33, 404–415. 20371137 10.1016/j.neurobiolaging.2010.02.018

[fsn31523-bib-0027] Soliman, K. M. (2001). Changes in concentration of pesticide residues in potatoes during washing and home preparation. Food and Chemical Toxicology, 39, 887–891. 11434996 10.1016/s0278-6915(00)00177-0

[fsn31523-bib-0028] Suwanchaichinda, P. , Khamkong, L. W. , & Satayavivad, J. (2005). Deltamethrin exposure affects host resistance to Plasmodium infection in mice. Environmental Toxicology and Pharmacology, 20, 77–82. 21783571 10.1016/j.etap.2004.10.004

[fsn31523-bib-0029] Uygun, U. , Koksel, H. , & Atli, A. (2005). Residue levels of malathion and its metabolites and fenitrothion in post‐harvest treated wheat during storage, milling and baking. Food Chemistry, 92, 643–647.

[fsn31523-bib-0030] Uygun, U. , Senoz, B. , & Hamit, K. (2008). Dissipation of organophosphorus pesticides in wheat during pasta processing. Food Chemistry, 109, 355–360. 26003358 10.1016/j.foodchem.2007.12.048

[fsn31523-bib-0031] Uygun, U. , Senoz, B. , Öztürk, S. , & Koksel, H. (2009). Degradation of organophosphorus pesticides in wheat during cookie processing. Food Chemistry, 117, 261–264. 10.1016/j.foodchem.2007.12.048 26003358

[fsn31523-bib-0032] Zabrodskii, P. F. , & Germanchuk, V. G. (2001). Role of activation of the sympathoadrenal system in the realization of immune reactions during acute poisoning with organophosphorus compounds. Bulletin of Experimental Biology and Medicine, 132, 966–968. 11782794 10.1023/a:1013667227881

[fsn31523-bib-0033] Zhao, P. Y. , Huang, B. Y. , Li, Y. J. , Han, Y. T. , Zou, N. , Gu, K. J. , … Pan, C. P. (2014). Rapid multiplug filtration cleanup with multiple‐walled carbon nanotubes and gas chromatography‐triple‐quadruple mass spectrometry detection for 186 pesticide residues in tomato and tomato products. Journal of Agriculture and Food Chemistry, 62, 3710–3725. 10.1021/jf405240j 24512455

[fsn31523-bib-0034] Zhou, J. H. , & Jin, S. S. (2009). Safety of vegetables and the use of pesticides by farmers in China: Evidence from Zhejiang province. Food Control, 20, 1043–1048.

[fsn31523-bib-0035] Zhou, X. W. , Liu, H. F. , & Zhao, X. H. (2015). The potencies of three microorganisms to dissipate four organophosphorus pesticides in three food materials during traditional fermentation. Journal of Food Science and Technology, 52, 7353–7360.

[fsn31523-bib-0036] Zhu, F. (2014). Influence of ingredients and chemical components on the quality of Chinese steamed bread. Food Chemistry, 163, 154–162. 24912711 10.1016/j.foodchem.2014.04.067

